# Development and psychometric evaluation of the Early Period Mother–Infant Bonding Indicators Assessment Scale

**DOI:** 10.1590/1806-9282.20241379

**Published:** 2025-06-02

**Authors:** Ozge Karakaya Suzan, Nursan Cinar

**Affiliations:** 1 Sakarya University, Institute of Health Sciences, Department of Nursing – Sakarya, Turkey.; 2 Sakarya University, Faculty of Health Sciences, Department of Nursing – Sakarya, Turkey.

**Keywords:** Mother, Infant, Mother-child relations, Reliability, Validity

## Abstract

**OBJECTIVE:**

The aim of this study is to develop the Early Period Mother–Infant Bonding Indicators Assessment Scale and to examine its validity and reliability.

**METHODS:**

This is a cross-sectional, methodological, correlational study. This study was carried out with 143 mothers in the Sakarya Training and Research Hospital Maternity Service between February and April of 2021. The content validity of the scale was assessed by consulting 11 experts. Exploratory and confirmatory factor analyses were performed, and the tool's internal consistency and construct validity were analyzed.

**RESULTS:**

For the mothers, the average age was 28.19±5.71. The Cronbach's alpha coefficient for the overall scale was 0.83, and the Cronbach's alpha values for the subscales were 0.85–0.80. The correlations between the physical and emotional intimacy and exploration and striving subdimensions with the whole scale were found to be highly positive and statistically significant (r=0.919, r=0.869, p<0.005). The exploratory factor analysis showed that the scale explained 55.59% of the total variance. The confirmatory factor analysis found that the physical and emotional intimacy and exploration and striving subdimension factor loads ranged from 0.53 to 0.75 and 0.59 to 0.77, respectively.

**CONCLUSION:**

The two-factor, 13-item Early Period Mother–Infant Bonding Indicators Assessment Scale was confirmed to have sufficient reliability and validity. With these measurement tools, mothers’ behaviors towards mother–infant bonding right after birth will be evaluated, and mothers will be directed to get support in the area of weakness.

## INTRODUCTION

Mother–infant attachment is defined as a loving, emotional, warm, and close relationship between the mother and the child^1^. The relationship between the mother and the child, established at the early stages of life, is crucial since it functions as a prototype for different relationships at later stages of an individual's life. The number of studies on mother–infant attachment has recently increased^2,3^. In our research, the term bonding is used with the objective of describing the mother's feelings toward her infant, and it differs from attachment, involving the infant's behavior toward the mother. Maternal–infant bonding, which is thought to begin before birth^4^ progresses based on interaction during and after birth. Early mother–infant bonding may impact children's postnatal social, emotional, and cognitive development^5^. Hence, it is important to understand the nature of the mother–infant bond and the factors affecting it during pregnancy and the early postpartum period.

Since the 1970s, measurement tools have begun to be developed, which allow assessing the level of mother–infant bonding. The Maternal Attachment Scale developed by Muller^6^ and available in Turkish, Japanese, Dutch, Korean, Chinese, and Indonesian versions is one of the tools for measuring mother–infant bonding. The Maternal Attachment Scale is used after the postpartum first month and measures maternal behaviors in the form of a situational question list. However, mother–infant bonding develops during pregnancy, childbirth, and the postpartum period. Especially the early postpartum period represents the most important time for bonding. The time right after birth is a sensitive period. Hence, this period is important for enhancing mother–infant bonding. Klaus and Kennell reported that the first minutes after birth are a critical period for a mother's bonding with her infant. This process is usually most intense in the first 72 h after birth^7^. The mother, who begins to recognize and perceive her infant before birth, tries to discover him/her in the first minutes after the infant is born^8^. She examines the infant's face, hands, and other parts of the body, tries to establish eye contact with the infant, and talks to the infant. In the following hours, she interprets the infant's reactions and behaviors and tries to make sense of them. These behaviors strengthen mother–infant bonding. Researchers have shown that the newborn establishes eye contact and sees, hears, and reacts by imitating facial expressions, even acting in harmony with the mother's voice, in the first minutes and hours of life^9^. Skin-to-skin contact, kangaroo care, and breastfeeding in the first hour of life, hugging, mother sharing the same room with infant, touching, massage, music therapy also contribute to bonding^10^. Hence, this period is important for enhancing mother–infant bonding. Furthermore, in the evaluations of mother–infant bonding, the inability of mothers to express themselves or the observational determination of unexpressed bonding difficulties by healthcare professionals reveals the importance and need for developing a scale based on this observation that can be applied immediately after birth. The purpose of our study is to develop the Early Period Mother–Infant Bonding Indicators Assessment Scale and examine its validity and reliability.

## METHODS

### Study design

The authors designed the current study as cross-sectional, methodological, descriptive, and correlational.

### Sample/participants

Our research was conducted in the maternity ward of Sakarya Training and Research Hospital Training and Research Hospital. Researchers state that the number of samples should be at least five times the number of scale items, and ideally 10 times the number of items, during a scale's development^11^. The study was conducted with a total of 143 mothers over 18 years of age, volunteering to participate in the research, having no newborn-related health problems, being the mother, of a single infant, the infant being born at term, the mother not being pregnant with assisted reproductive technology.

### Data collection tools

#### Sociodemographic form

The above-mentioned form was filled out by the mothers during their hospital stay. It includes 26 questions.

#### Early Period Mother–Infant Bonding Indicators Assessment Scale

##### Phase I: Preparation and scoping phase

Extensive searches were done in the literature to understand mother–infant bonding behaviors^6^, indicators showing mother–infant compliance^12^, and sensitive parental behaviors^1^. Moreover, scales measuring mother–infant attachment in the literature were examined^13^.

##### Phase II: Development of the questionnaire items and rating scale

The scale includes 13 items demonstrating physical and emotional intimacy, exploration, and striving. The best practices for creating items were followed^14^. The user observes the mother–infant bonding indicators and marks the scale items accordingly in the weak, moderate, or good categories. For each item, poor is scored as zero (0) points, moderate as one (1) point, and good as two (2) points. Expert opinions were utilized to finalize the draft scale.

##### Phase III: Feedback from experts (content validity)

An e-mail was sent to Pediatric Nurse (n:6), Pediatrician (n:2), Obstetrics and Gynecology Specialist (n:1), Psychiatric Nursing (n:1), Psychiatrist (n:1). All experts were researchers with clinical experience and specialization in developing mother–infant bonding. A draft of the Early Period Mother–Infant Bonding Indicators Assessment Scale and a content validity index (CVI) rating form were sent to the research experts, who were asked to rate the relevance of every item. The rating of the relevance was performed on a three-point scale in the following way: 1=not at all relevant, 2=need to be fixed, and 3=highly relevant. The revision of two scale items was performed by analyzing the completed CVI forms and the comments of the research experts^15^.

##### Phase IV: Pilot study

After verifying the scale's contents, a pilot study was carried out with a 13-item draft scale. Face validity was tested with five observer nurses applying the pilot study to 10 mothers. The Item-based Face Validity Index varies between 0.80 and 1.00. The Face Validity Index for the overall scale was found to be 0.97.

#### Psychometric evaluation

The evaluation of internal consistency and construct validity was performed based on the data acquired from the pilot study. Internal consistency was evaluated by Cronbach's alpha (α)^15^. The values vary between 0 and 1, and a score of 0.7 or higher is usually considered to indicate good internal consistency. Exploratory factor analysis (EFA) and reliability analysis were performed.

### Data collection

Our research was conducted between February and April 2021. The observation of each mother admitted to the maternity ward began in the mother's room at least 2 h after hospitalization. The observers were academic nurses who had received doctoral training in pediatric nursing and worked in the maternity ward. Two observers independently observed the mother–infant interaction for 107.52±31.82 and 107.66±31.54 min simultaneously, in line with the items of the Early Period Mother–Infant Bonding Indicators Assessment Scale, and independently recorded their observations. While making the observations, the observers went to the mothers at the same time intervals and began to observe the behavior of the mother and the infant. Later, the descriptive information questionnaire was completed by conducting face-to-face interviews with the mothers.

### Data analyses

Data analysis was conducted in IBM SPSS Statistics for Windows, version 26 (IBM Corp.). Descriptive statistics were presented as frequency, percentages, means, and standard deviation (SD). In the reliability analysis, item-total score analysis, Cronbach's alpha coefficient, and interobserver agreement analysis were conducted with the objective of determining the scale's and subscales’ internal consistency. In the validity analysis, CVI, EFA, and confirmatory factor analysis (CFA) were carried out to reveal validity. Statistical significance was accepted at p<0.05.

### Ethical considerations

Ethical approval was received from the Health Ethics Committee of Health Ethics Committee of Sakarya (Date: 22.12.2020 Issue: E-16214662-050.01.04-6787-08), whereas institutional permission was acquired from the hospital where the research was done. Prior to initiating the research, the mothers were told that they would be informed about the study by the researchers and that their personal information would be protected. Verbal and written consent was acquired following the principle of voluntariness.

## RESULTS

### Demographic data

A total of 143 mothers participated in our study. Their mean age was 28.19±5.71 years. Of the total sample, 48.9% (n=70) were elementary school graduates, 76.9% (n=110) were nuclear family, 81.8% (n=117) were housewives, 91.8% (n=131) had not received parenting preparation training, and 83.9% (n=120) were multiparous.

The Early Period Mother–Infant Bonding Indicators Scale has 13 items. The minimum and maximum scores on the said scale are 0 (13×0) and 26 (13×2), respectively. Increased levels of mother–infant bonding produce higher scores on the scale. The scale has two subscales. The physical and emotional intimacy subscale consists of eight items, while the exploration and striving subscale consists of five items. The scale can be applied to the mother and the infant after birth during their hospital stay.

### Content validation

The item-based content validity rate changed in the range of 0.63–1.00, with a CVI value of 0.81. Each item's CVI was >0.67, the scale was found to be statistically significant.

#### Construct validity

Exploratory factor analysis (EFA): Table 1 contains the EFA findings for the Early Period Mother–Infant Bonding Indicators Assessment Scale. The results revealed that the Kaiser-Meyer-Olkin (KMO) coefficient was 0.87, and Bartlett's test result was χ^2^=816.323, df=78, p<0.001. Two factors with an eigenvalue above 1.00 were found, explaining 55.89% of the total variance. The *physical and emotional intimacy* subscale explained 30.94% of the total variance, while the *exploring and striving* subscale explained 24.95% of the total variance. According to the EFA results, the factor loadings of the *physical and emotional intimacy* and *exploration and striving* subscales varied between 0.54–0.86 and 0.65–0.77, respectively (Table 1). When the scree plot results were examined, it was appropriate for the scale to be two-dimensional. However, it was checked with Monte Carlo Parallel Analysis, and when we compared the eigenvalues, it was seen that only two factors were above the limit. Since CFA is compatible with EFA, the scale structure did not change as a result of CFA.

**Table 1:** Exploratory factor analysis, factor loading values of the items on the Early Period Mother–Infant Bonding Indicators Assessment Scale, the principal component analysis, and the rate of variance explained by factors (n=143).

Subscales	Item number	Factor loading values in principal component analysis	Eigenvalues	Rate of variances explained by factors
Physical and emotional intimacy subscales	Item 1	0.861	4,023	30,943
	Item 2	0.787		
	Item 11	0.689		
	Item 13	0.682		
	Item 12	0.656		
	Item 3	0.607		
	Item 4	0.557		
	Item 8	0.549		
Exploring and striving subscales	Item 7	0.773	3,244	24,951
	Item 10	0.762		
	Item 5	0.719		
	Item 6	0.651		
	Item 9	0.650		
Total scale				55,894

**Figure 1:**
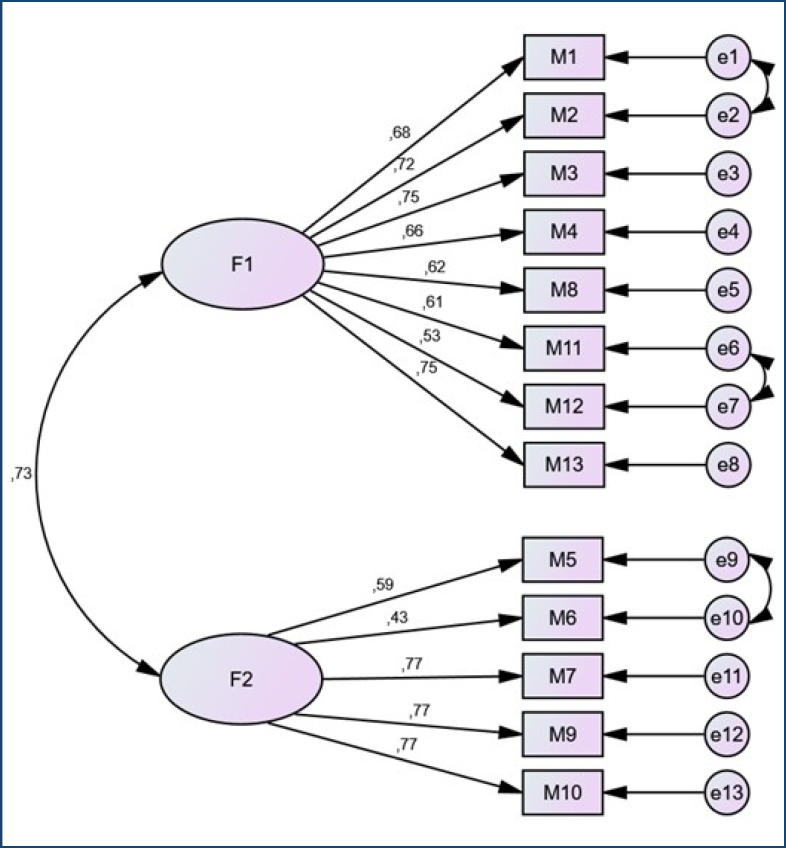
Confirmatory factor analysis of the Early Period Mother–Infant Bonding Indicators Assessment Scale (n=143).

Confirmatory factor analysis (CFA): CFA was used to confirm the two dimensions of the instrument obtained by the EFA. CFA results of the Early Period Mother–Infant Bonding Indicators Assessment Scale. CFA revealed that the *physical and emotional intimacy* and *exploration and striving* subscale factor loadings ranged from 0.53 to 0.75 and 0.59 to 0.77, respectively. The fit indices of the Early Period Mother–Infant Bonding Indicators Assessment Scale were as follows: Root Mean Square Error of Approximation (RMSEA)=0.079, χ^2^/df=1.89, Tucker-Lewis Index (TFI)=0.91, Incremental Fit Index (IFI)=0.93, Comparitive Fit Index (CFI)=0.92, and Standardized Root Mean Square Residual (SRMR)=0.060 (Figure 1).

### Reliability analysis

#### Internal consistency analysis

We found the reliability coefficient of the Early Period Mother–Infant Bonding Indicators Assessment Scale (13 items) as α=0.83. The reliability coefficients of the *physical and emotional intimacy* (eight items) and *exploration and striving* (five items) subscales were α=0.85 and α=0.80, respectively. The reliability analysis examined the item-total score correlations of the 13-item scale. It was observed that the correlation coefficients of the scale items with the whole scale varied between 0.390 and 0.691 (see Table 2). McDonald's Omega value was found to be 0.871 for the first sub-dimension, 0.837 for the second sub-dimension, and 0.922 for all items.

**Table 2:** Factor analysis results of the Early Period Mother–Infant Bonding Indicators Assessment Scale substances (n=143).

	Subscales	Item number	Item total score correlations (n=143)	Cronbach's alpha		McDonald's Omega
The Early Period Mother–Infant Bonding Indicators Assessment Scale	Physical and emotional intimacy subscales	Item 1	0.561	0.851	0.882	0.871
		Item 2	0.630			
		Item 3	0.691			
		Item 4	0.633			
		Item 8	0.548			
		Item 11	0.571			
		Item 12	0.464			
		Item 13	0.653			
	Exploring and striving subscales	Item 5	0.545	0.803		0.837
		Item 6	0.390			
		Item 7	0.638			
		Item 9	0.666			
		Item 10	0.600			

#### Item-total score correlations and subscale correlations

The correlations of the subscales with the whole scale were also studied. As a result of the applied Pearson correlation analysis, the correlations between the *physical and emotional intimacy* and *exploration and striving* subscales with the whole scale were found to be highly positive and statistically significant (r=0.919, r=0.869, p<0.005) (Table 3). In the CFA model, the relationship level between the sub-dimensions was determined as r=0.73, p=0.000.

#### Interobserver agreement

Intraclass correlation coefficient (ICC) analysis was used to determine agreement between the observers. ICC values of 0.997 and 0.998 were found for the *physical and emotional intimacy* and *exploration and striving* subscales, respectively. An ICC value of 0.998 was found for the scale.

## DISCUSSION

We examined the scales in the literature that assess mother–infant bonding^1,13^. There are few scales that evaluate the mother's bonding behaviors to her infant immediately after birth; it is important to develop the Early Period Mother–Infant Bonding Indicators Assessment Scale.

### Scale validity

Factor analysis is essential in scale development^14^. The EFA results revealed that the KMO coefficient was 0.87, and Bartlett's test result was significant (p<0.001), demonstrating that the sample size was suitable for factor analysis and the data were homogeneously distributed. A structure containing two factors—*physical and emotional intimacy* and *exploring and striving*—with an eigenvalue above 1.00 that explained 55.89% of the total variance was found. Our results suggest that the scale items adequately explain the variance and provide evidence of construct validity^16^. According to the EFA results, the factor loadings of all items on the scale were above 0.50. The items on the scale had medium and high factor loading values, which indicate that the items explain sufficient variance and show that the construct validity is acceptable.

CFA revealed that the factor loadings in the subscales of the Early Period Mother–Infant Bonding Indicators Assessment Scale were higher than 0.50. RMSEA was <0.080. The indicated values demonstrated that the data fit the model, verified that the two-factor structure, scale items, and subscales were related to the scale, and proved that the items in every subscale adequately determined their factors. Our findings prove that the scale has structural validity and is a valid tool^17^.

### Factor 1: Physical and emotional intimacy and Factor 2: Exploring and striving

Researchers have stated that the physical and emotional intimacy^18–23^ and exploring and striving^1,21,24^ of the mother support mother–infant bonding and increase interaction with the infant. Cronbach's alpha coefficient for the factor in question was determined to be >0.7.

### Scale reliability

Cronbach's alpha value, which measures the scale's reliability, was 0.83 for the overall scale. Cronbach's alpha coefficient for each factor was >0.70. The item-total score correlations, subscale correlation coefficients, and scores for the overall scale and all subscales were positive, statistically significant, and higher than 0.30. According to the presented findings, all items of the scale have a sufficient correlation with the subscale total score, the item reliability of the subscales is high, and the newly developed scale is reliable.

The item-total score correlation coefficients of the 13 items ranged from 0.39 to 0.69. According to the ICC analysis results applied to determine the agreement between the observers, ICC values of 0.997 and 0.998 were found for the *physical and emotional intimacy* and *exploration and striving* subscales. An ICC value of 0.998 was found for the Early Period Mother–Infant Bonding Indicators Assessment Scale. Our results support the scale's reliability and indicate that the interobserver agreement of this scale is high.

The item-based content validity rate changed in the range of 0.63–1.00, with a CVI value of 0.81. Since the content validity rate of the scale was above 0.54 and each item had a CVI>0.67, 13 items remained on the scale.

## CONCLUSION

The Early Period Mother–Infant Bonding Indicators Assessment Scale is a valid and reliable measurement tool developed to assess mother–infant bonding during the postpartum period.
